# The Effects of a Recollection-Based Occupational Therapy Program of Alzheimer's Disease: A Randomized Controlled Trial

**DOI:** 10.1155/2020/6305727

**Published:** 2020-07-30

**Authors:** DeokJu Kim

**Affiliations:** Department of Occupational Therapy, Cheongju University, Daesung-ro, 298, Cheongwon-gu, Cheongju-si, Chungcheongbuk-do, Republic of Korea

## Abstract

Considering the high socioeconomic costs related to the increasing number of dementia patients and their poor quality of life and that of their families, it is important to identify the condition early on and provide an appropriate intervention. This study organized a recollection-based occupational therapy program: a nonpharmacological intervention consisting of five categories of activities (physical, horticultural, musical, art, and instrumental activity of daily living; IADL) and applied it to those having a mild stage of Alzheimer's disease. The experimental group participated in a total of 24 sessions––five times per week for one hour per session––while the control group took part in regular activities offered by the existing facilities. The experimental group presented improved cognitive functions, reduced depression, and enhanced quality of life; the two groups showed a statistically significant difference in every category. This study is meaningful in that it made a cognitive stimulation program concerning five different categories, implemented it for people suffering mild dementia, and confirmed positive outcomes. If a systemic version of the program is offered in dementia care facilities, it is expected to make a considerable contribution to the care of dementia patients.

## 1. Introduction

Population aging has become a grave social issue globally. Korea, in particular, is seeing the fastest population aging among OECD member countries; it became an aged society in 2018 with the old accounting for 14.3% of the entire population and is estimated to enter a superaged society in 2026 with the figure reaching 20.8% [1]. Physical function, along with cognitive abilities (including memory), deteriorates with age. In particular, Alzheimer's disease (AD) poses as a serious threat for overall cognitive function [2]. According to reports from the Korean National Institute of Dementia, the prevalence of AD has sharply increased as the population has aged, with a national estimate of approximately 75,000 AD patients aged over 65 in 2018, making up about one-tenth of the overall senior population [3].

Alzheimer's disease (AD) is the most frequent cause of dementia in the older population. AD consists of progressive cognitive decline, frequently presenting initially as short-term memory impairment and affecting judgment, decision making, and orientation skills. Later stages of the disease also present with behavioral disturbances and language abnormalities [4]. Socioeconomic costs following an increase in AD should be reduced, and the prevalence of AD must be decreased through early diagnosis and treatment so as to improve the quality of life of patients and caregivers. In order to do so, social support measures should be in place. Nonpharmacological interventions are most frequently used to prevent and treat AD. Pharmacological intervention helps slow the progress of AD and partially releases cognitive and mental behavioral symptoms, yet it largely preserves the existing symptoms and its side effects, which may occur after taking the medicine for a long period of time, can make AD patients feel frustrated, and can leave their family anxious and helpless [5]. Hence, nonpharmacological interventions are attracting more attention as preventive and therapeutic measures. They include cognitive stimulation activities, which help people live independently while maintaining a high quality of life; improve cognitive, physical, and sensory functions; and develop social skills through various activities and programs [6]. Meanwhile, AD patients are currently participating in activities at home or in adult daycare centers that are randomly selected by program instructors. Such activities, however, present some issues. Face-to-face cognitive stimulation training provides a good example. While there is a booklet that introduces the training, detailed explanation about how it works are missing. Moreover, the training itself mostly consists of written tasks, so it is ineffective in encouraging physical movement. As for computerized cognitive training, it cannot be offered when there is no computer program. In this respect, a systemically organized cognitive program should be provided that addresses those drawbacks.

Depression is one of the most prevalent psychological diseases in old age, along with the increased incidence of AD. The onset of depression can occur in the early stages of cognitive dysfunction and has been reported to increase the risk of AD by increasing the rate of progression of cognitive dysfunction. As such, depression may be a prodromal symptom or may increase the rate of the onset of AD [7]. One of the positive functions of recollection is that it reinterprets and reunifies the unresolved matters of the past, resolves psychological conflict, cognitively reconstructs various senses of defeat, and gives new meaning to life, thereby helping overcome a depressing reality and boosting self-integration [8]. Recollection activities recover forgotten personal experiences and memories, allowing the subject to experience emotions linked to past experiences. Familiar objects, media (such as pictures), and songs can be used for recollection [9]. It is also an activity of locating oneself in the present by using the intact long-term memory of the past and is rarely affected by the subject's education or severity of the symptoms [10]. Previous studies mostly depended on story-telling, films, images, and picture books for recollection activities, but some reports suggest that a recollection method accompanied by occupational activity is more effective than using just auditory or visual stimuli, and a program focused on activities that the subject used to perform in the past is better in terms of boosting memory, self-esteem, and interaction [11, 12].

Taking these factors into consideration, the researchers referred to the AD prevention program offered by local dementia support centers and adult daycare centers in Seoul [13, 14]. In an effort to determine the efficacy of reconstructed occupational therapy programs, these programs integrated recollection throughout the five categories of activities identified in previous research as being beneficial to cognitive function (physical, horticultural, musical, artistic, and IADL).

## 2. Methods

### 2.1. Participants

This study examined AD patients with a mild stage of AD who visited A and B adult daycare center located in P city from February to March, 2019. The detailed recruitment criteria were as follows: (1) seniors aged 65 or older. (2) those who did not have a brain disorder other than dementia, (3) those who were diagnosed with mild AD, (4) those who were able to follow instructions and did not have any auditory or visual impairment, and (5) those who were informed of the purpose and the methods of the study and agreed to participate. The exclusion criteria were as follows: (1) those who had difficulty in identifying objects due to poor eye sight or a visual perception issue, (2) those who had a serious physical disorder, (3) those who suffered a brain injury apart from dementia, and (4) those who were unable to concentrate on an activity because of an unstable vital sign.

### 2.2. Study Design

**Figure 1:**
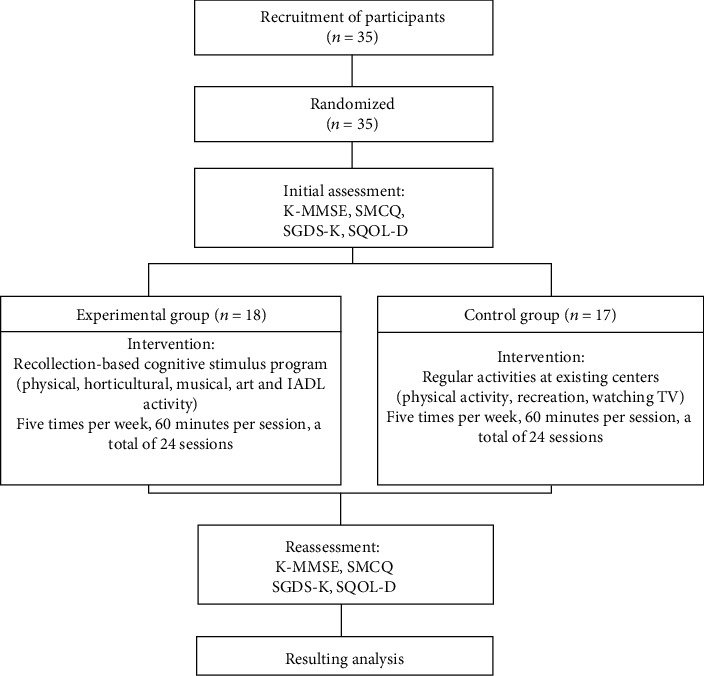
Study process.

For this study, 35 dementia patients with a mild stage of AD who were attending an adult daycare center were recruited and then randomly divided into the experimental and control groups. The majority of the users for both institutions were AD patients, as were the selected subjects. Before the intervention, the two groups' general characteristics and cognitive functions were first examined to confirm homogeneity. All assessments and interventions were performed by a therapist who had over five years of occupational therapy experience. The experimental group joined a recollection-based occupational therapy program constructed by the author where they were asked to engage in one activity every day from Monday to Friday. By contrast, the control group participated in the regular activities provided by their existing daycare centers. Regular activities included physical and recreational activities, arts and crafts, music activities, and rest and are listed in detail in Table 1. The program for the experimental group was offered for a total of 24 sessions, five times per week for an hour per session, and the initial evaluation and reevaluation were conducted prior to and after the intervention, respectively. The program was offered in both institutions, but at different times in order to avoid overlap. The process is depicted in Figure 1.

### 2.3. Recollection-Based Occupational Therapy Program

The program was developed by referring to dementia prevention programs that were actually provided in dementia support centers and community welfare centers in Seoul. Activities that many studies found effective in preventing dementia through improved cognitive functions were divided into five categories and adopted to the program developed [15–17]. The program was made over a year, from January 2018 to December 2018. Expert advice was sought from “an occupational therapist with at least five years of experience in senior occupational therapy” when selecting the final program to adopt in the study. As previous research identified the need for at least 10 experts for the Delphi technique [18], 10 experts were consulted using the Delphi survey. Initially, 20 programs for each of the “physical, horticultural, musical, artistic, and IADL” categories of activities were made for a total of 100 programs. Further, nine programs for each activity, resulting in a total of 45 programs, were finalized following three sessions of expert consultation. Each program was further divided by content into the recollection of childhood, adulthood, and late adulthood. As a reference for the program instructor, a manual for the entire program was produced that contained images of what to prepare for in each session and how activities should be done consecutively to offer detailed information on which functions could be improved through such activities (see Table 2).

### 2.4. Measurements

#### 2.4.1. Functional Independent Measure (FIM)

This well-known assessment tool was developed in 1983 by Ganger et al. for objective functional assessments of ADLs. Each domain is rated by a score ranging between 1 and 7, depending on the level of independence, with a score of 1 indicating total dependence, and where a score of 7 indicates total independence. The assessed domains include self-care, sphincter control, transfer, locomotion, communication, and social cognition. Achievable scores range from 18 to 126, with higher scores indicating higher ADL function [19].

#### 2.4.2. Korean Mini-Mental Status Examination (K-MMSE)

This is an assessment tool that has been translated into Korean and standardized by Kang et al. [20]. It contains a total of six categories and 27 items consisting of orientation to time, orientation to place, registration, attention and calculation, recall, language, and visuospatial composition ability. The maximum score is 30, with scores higher than 24 indicating normal, 20–23 points indicating mild cognitive impairment, 10–19 points indicating moderate cognitive impairment, and scores of 9 or less indicating severe cognitive impairment [21].

#### 2.4.3. Subjective Memory Complaints Questionnaire (SMCQ)

This questionnaire has 14 items and is designed to evaluate old people's subjective memory deterioration. All items are rated on a 2-point scale of either “yes” or “no,” and the optimal articulation point for dementia is 5/6. The factors are divided into three types: overall, everyday life, and subjective memory complaints [22].

#### 2.4.4. Short-Form Geriatric Depression Scale-K (SGDS-K)

While the original Geriatric Depression Scale (GDS) was developed by Yesavage et al. [23] as a 30-item self-report depression scale, it was later simplified to a 15-item scale (SGDS) by Sheikh and Yesavage [24] as it took too much time to fill in the 30 items. In this study, the 15-item SGDS-K, the Korean version of SGDS translated and standardized by Cho et al. [25] was employed to measure the participants' depression. With an articulation point of 8, this tool is widely used by local communities when identifying depression disorders.

#### 2.4.5. Geriatric Quality of Life-Dementia (GQOL-D)

This tool consists of 15 items––13 on dementia patients' physical health, psychological health, social relationships, and environment; one on overall health; and one on life satisfaction––and is useful in evaluating dementia sufferers' quality of life [26].

### 2.5. Data Analysis

For statistical analysis, the program SPSS window version 23.0 was used. The significance level was set at *p* < 0.05. General characteristics of the participants were analyzed for frequency using descriptive statistics. Paired and independent *t*-tests were carried out to compare the two groups pre-and postintervention and to compare intergroup differences, respectively.

## 3. Results

### 3.1. Demographics and Baseline Characteristics

The total of 35 participants consisted of nine men and 26 women. The experimental group was 80.6 ± 5.12 years old on average, while the control group was 77.88 ± 5.49. Educational years of the experimental group was 6.62 ± 3.64 and that of the control group was 7.19 ± 3.48. Functional Independence Measure (FIM) scores of the experimental group was 78.82 ± 11.42; the control group registered scores of 79.27 ± 12.98. In terms of demographic characteristics, the two groups presented no statistically significant difference, thereby confirming homogeneity (Table 3).

### 3.2. Test for Homogeneity in Preintervention Cognitive Functions, Depression, and Quality of Life

Before the intervention, the homogeneity of the experimental and control groups was tested in terms of cognitive functions, depression, and quality of life. There was no statistically significant difference between the groups, thus proving the homogeneity (Table 4).

### 3.3. Comparison before and after Recollection-Based Occupational Therapy Program

The results of the ex-ante and ex-post analysis of recollection-based occupational therapy program as well as the comparison of the experimental and control groups are as follows. In terms of subjective memory deterioration measured by SMCQ, the experimental group's average score went from 5.83 ± 3.68 to 4.16 ± 2.09 after the program, presenting a statistically significant improvement (*p* < 0.05), whereas the control group showed no significant change. When the postintervention outcomes of the experimental and control groups were compared, there was a significant difference between the two groups (*p* < 0.05). Meanwhile, concerning changes in cognitive functions examined via K-MMSE, the experimental group, who scored 18.70 ± 1.68 before the program, afterwards scored 19.56 ± 2.17, for a statistically significant change (*p* < 0.05), but such improvement was not observed in the control group. The post hoc comparison showed a significant gap between the two groups (*p* < 0.01).

Regarding changes of depression measured via SGDS-K, depression of the experimental group decreased significantly from 6.55 ± 3.50 before the program to 4.10 ± 3.66 after (*p* < 0.05), whereas there was no significant change in the control group. When compared after the program, the experimental and control groups presented a significant difference from each other (*p* < 0.05). Finally, with regard to changes of quality of life assessed via GQOL-D, there was a statistically significant improvement for the experimental group as their score rose from 30.11 ± 7.06 to 33.50 ± 7.22 after the intervention (*p* < 0.01), but such change was not found in the control group. The post hoc comparison suggested a significant difference between the two groups (*p* < 0.05). Of the cognitive functions, depression and quality of life presented the biggest changes in the quality of life to the participants (Table 5).

## 4. Discussion

In this study, recollection-based occupational therapy program was reconstructed to apply to mild AD patients and its effects were investigated. The program was made up of five-category activities of physical, horticultural, musical, art, and ADL, and each category used themes whereby the subjects could recall their childhood, adulthood, and late middle age. After about a year of composition, it is then applied to AD patients to assess its effects.

To investigate the program's effects, both SMCQ and K-MMSE were used, which showed that the experimental group showed a statistically significant reduction in SMCQ compared to the control group and also showed improvement in K-MMSE. This corresponds to the results of previous research that found recollection is effective in maintaining and improving AD patients' cognitive functions because it helps in clarifying blurred memories [26]. Moreover, the improved cognitive functions observed in this study seem attributable to the fact that the participants engaged in the program more actively as they recalled the past and engaged in activities that were familiar to them; in the process, they also utilized procedural memories remaining in their long-term memory, thereby revitalizing the brain and encouraging active participation [27].

The experimental group also showed reduced levels of depression after the program, and according to Eom [28], senior citizens under a group recollection therapy can alleviate distress and experience feelings of support by sharing their memories with others; they also experience a positive change in emotions by bringing good emotions of the past to the present. Geriatric depression is characterized by losing interest in one's surroundings, frequently feeling fatigue, having difficulty in waking up early in the morning, and often feeling helpless about death [29]. In this study, however, AD patients presented a significant decrease in depression after participating in the recollection-based cognitive stimulation program and are expected to regain vitality in daily living.

This study found that the recollection-based occupational therapy program is effective in boosting quality of life as well. This is because the therapy helps AD patients not only improve their cognitive functions but also speak and make nonverbal expressions more frequently; alleviating problematic behavior, feel stable emotions, and increase social exchange, thereby being able to lead to a better life [30]. It was found that the quality of life of people suffering dementia is significantly related to negative emotions like anxiety and depression, which have a tremendous impact on their mental status [31]. Butler [32] claimed that people who recall their past and look back on their life can make an assessment of how they have lived and thereby find new value in living. In this sense, it seems that the recollection program used in this study gave the participants an opportunity to remember the good old days while enjoying fun activities. By taking part in activities linked to different life stages––childhood, adulthood, and late middle age––and sharing common experiences with others from the same generation, the participants formed greater bonds with each other, improved social skills, and as a result, felt better about their lives.

The recollection-based occupational therapy program consists of art, music, and horticultural activities where the subjects can make a piece of art or enrich their emotions through music, as well as engage in physical activities. One of the merits of art activities is that they reinforce the subjects' visuospatial aspects and induce positive emotions [26]. By participating in art activities, AD patients, who have deteriorated physical and cognitive functions and thus are unable to articulate their emotions, can release energy and express their emotions or thoughts while reducing defensiveness or self-control [33]. It has been proven that horticultural activities are highly effective in promoting positive thoughts, refreshing the mood, easing stress, and stabilizing emotions by having the subjects take care of plants thereby stimulating the five senses [34]. This is why horticultural therapy is being adopted by many facilities for AD patients these days. As for ADL, daily life activities that AD patients usually do at home were reconstructed for the program. This was because they tend to feel less pleasure and satisfaction with themselves when performing unfamiliar activities [35]. By participating in ADL that they were familiar with and good at in their daily lives, the participants in this study experienced a sense of their own existence and self-efficacy, thereby becoming pleased and satisfied.

This study is not without limitations. First, it is difficult to generalize the results to the general public as this study examined a small number of people. Moreover, there was no follow-up inspection to check whether the participants maintained the improved functions after the program. Despite these limitations, there is significance in the fact that programs involving the five activities—which were proven to be effective for AD patients—were reconstructed throughout this study and that positive outcomes were obtained from successful real-life applications of the programs to AD patients.

## 5. Conclusion

This study organized recollection-based occupational therapy program and applied it to dementia patients suffering mild AD to investigate its effect. It was found that the intervention was effective in improving cognitive functions, reducing depression, and enhancing the patients' quality of life. If a more systemic and organized program is introduced in long-term care facilities and dementia relief centers, it is expected to make a great contribution in terms of policymaking.

## Figures and Tables

**Table 1:** Typical activities at the adult day care center (provided to the control group).

Category	Task	Examples of activities
Physical activity	Strength training	Raises using light weights, knee bends
Physical activity	Balance exercise	Balancing on one leg without support, maintaining standing position on a balance board
Physical activity	Flexibility training	Upper- and lower-body stretches
Physical activity	Cardiovascular exercise	Walking a set distance
Recreation	Games	Throwing darts, balloon volleyball, bowling, Yut-Nori (folk game)
Arts and crafts	Hands-on creations	Origami, clay art, knitting
Musical activity	Folk sing alongs	Singing along folk songs
Rest	Watching TV	TV program of choice

**Table 2:** Contents of the program (provided to the experimental group).

Category	Stage of life	Task	What to recall (or to do)	Difficulty
Physical activity	Childhood	(i) Make a Yut board and play Yut (ii) Make Tuho with newspaper and play Tuho (iii) Play cloth Jegichagi	(i) Memories of enjoying fun activities with family during holidays (ii) Memories of playing Tuho with friends in the town (iii) Memories of playing Jegichagi in the childhood	Easy
				Easy
				Easy
	Adolescence adulthood	(i) Play bowling with plastic bottles (ii) Play blue flag white flag game (iii) Play darts	(i) Memories of play bowling with co-workers and having fun (ii) Memories of attending a sports day at their child's school (iii) Memories of playing darts with their child	Medium
				Medium
				Hard
	Late middle age	(i) Play the fruit basket game (ii) Give massage (iii) Play the “dance with fun, then stop” game	(i) Memories of harvesting crops and fruits in the autumn (ii) Memories of getting massage from a grandchild (iii) Memories of dancing with a grandchild and having fun	Medium
				Medium
				Hard

Musical activity	Childhood	(i) Point to a body part (ii) Make a pipe out of a straw and play it (iii) Play the handbell	(i) Memories of pointing to a body part with friends and singing a song in childhood (ii) Memories of making a pipe with leaves found in the roadside and playing with it in childhood (iii) Play the handbell to an easy song such as “twinkle, twinkle, little star”	Easy
				Easy
				Medium
	Adolescence adulthood	(i) Sing a song associated with a given word (ii) Listen to the intro of a song and guess what song it is (iii) Beat time to traditional music	(i) Memories of songs that they enjoyed and sang often in adulthood (ii) Listen to the intro of a children's song that they used to sing for their child and give its correct title (iii) Memories of attending a town festival with their child and singing songs together	Medium
				Hard
				Hard
	Late middle age	(i) Sing songs in different beats and beat time (ii) Sing a song about hometown (iii) Listen to a traditional children's song and do a mask dance	(i) Sing along songs with 2/4, 3/3, and 4/4 beats, and beat time by lightly tapping their fingers on the table (ii) Sing a song that can help bring up memories of their hometown such as “spring in my hometown” or “Santa Lucia” (iii) Memories of dancing at a festival in town wearing a mask	Medium
				Medium
				Easy

Art activity	Childhood	(i) Draw a fingerprint (ii) Make a refrigerator magnet (iii) Make a crystal snowflake	(i) Memories of stamping a seal using their fingerprint in the childhood (ii) Remember animals or fruits they saw in the past (iii) Memories of having a snowball fight with friends in the childhood	Medium
				Medium
				Hard
	Adolescence adulthood	(i) Make a miniature folding screen (ii) Dye a cloth bag (iii) Make a household ledger	(i) Memories of preparing for ancestral rites (ii) Dye a bag while recalling memories of going grocery shopping with their child (iii) Make a household ledger while remembering younger days when they managed family finances	Hard
				Medium
				Medium
	Late middle age	(i) Draw and decorate the Korean flag (ii) Make a humidifier using felt cloth (iii) Dye a T-shirt	(i) Recall how Korea used to be in the past and compare it with the present (ii) Make a humidifier to protect their skin from getting drier while recalling younger days when they had good skin (iii) Decorate a T-shirt to give their grandchild as a gift	Easy
				Easy
				Easy

Horticultural activity	Childhood	(i) Make a name tag using pressed flower (ii) Make a frame using beans (iii) Dye fingernails with garden balsams	(i) Remember their name and find out who they are (ii) Remember their mother (iii) Memories of dying fingernails with garden balsams and running around in the field in childhood	Medium
				Medium
				Easy
	Adolescence adulthood	(i) Make a dish garden (ii) Make a topiary face (iii) Grow buds	(i) Memories of their family living in a small space harmoniously (ii) Memories of their youth (iii) Hard but fun memories of raising their child	Medium
				Hard
				Easy
	Late middle age	(i) Make a grass doll (ii) Make a potpourri (iii) Make a flower basket	(i) Recall what their grandchild looks like (ii) Memories of darning blankets and sewing in the past (iii) Memories of receiving flowers from their child as a gift	Medium
				Hard
				Easy

IADL activity	Childhood	(i) Play the chopstick game (ii) Make rice balls (iii) Do sand play	(i) Pick and move stuff using chopsticks while recalling the memories of learning how to use chopsticks in childhood (ii) Make rice balls while recalling ones they ate during the Korean war (iii) Memories of playing with earth with friends in childhood (feel what earth is like)	Hard
				Medium
				Medium
	Adolescence adulthood	(i) Hang the laundry (ii) Sew (iii) Flip holiday food	(i) Memories of washing their young child's clothes and hanging them on a clothesline (ii) Try sewing using a thick thread and felt cloth, and recall mending their child's clothes (iii) Try flipping through printed images of holiday food and recall preparing for holidays	Easy
				Medium
				Easy
	Late middle age	(i) Walk on a balance board (ii) Draw a rough map (iii) Make my own calendar	(i) Practice maintaining balance on a balance board (ii) Draw a rough map while recalling their current house and former houses (iii) Write down important family events	Medium
				Hard
				Easy

**Table 3:** Characteristic of participants.

	Experiment group (*n* = 18)	Control group (*n* = 17)	*p*
Gender, *n* (%)			
Male	3 (16.7)	6 (35.3)	0.223
Female	15 (83.3)	11 (64.7)	
Age (years)	80.6 ± 5.12	77.88 ± 5.49	0.138
Education (years)	6.62 ± 3.64	7.19 ± 3.48	0.211
FIM	78.82 ± 11.42	79.27 ± 12.98	0.237

FIM: Functional Independent Measure.

**Table 4:** Homogeneity test of dependent variables.

	Experiment group (*n* = 18)	Control group (*n* = 17)	*p*
SMCQ	5.83 ± 3.68	5.52 ± 2.03	0.802
K-MMSE	18.70 ± 1.68	18.10 ± 1.58	0.661
SGDS-K	6.55 ± 3.50	7.05 ± 3.30	0.665
GQOL-D	30.11 ± 7.06	28.64 ± 6.50	0.291

SMCQ: Subject Memory Complaint Questionnaire; K-MMSE: Korean Mini-Mental Status Examination; SGDS-K: Short-Form Geriatric Depression Scale-K; GQOL-D: Geriatric Quality of Life-Dementia.

**Table 5:** Change in outcome measurements after intervention.

	Pretest	Posttest	*p*
SMCQ			
Experiment group	5.83 ± 3.68^†^	4.16 ± 2.09^a∗^	0.012^b∗^
Control group	5.52 ± 2.03	6.05 ± 2.10	
K-MMSE			
Experiment group	18.70 ± 1.68	19.56 ± 2.17^∗^	0.005^∗∗^
Control group	18.10 ± 1.58	17.68 ± 1.64	
SGDS-K			
Experiment group	6.55 ± 3.50	4.10 ± 3.66^∗^	0.048^∗^
Control group	7.05 ± 3.30	7.05 ± 4.16	
GQOL-D			
Experiment group	30.11 ± 7.06	33.50 ± 7.22^∗^	0.003^∗∗^
Control group	28.64 ± 6.50	26.88 ± 4.68	

^†^Mean ± SD, ^∗^*p* < 0.05, ^∗∗^*p* < 0.01. ^a^A significant difference from baseline after intervention in each group using paired *t*-test. ^b^A significant difference between the 2 group using independent *t*-test. SMCQ: Subject Memory Complaint Questionnaire; K-MMSE: Korean Mini-Mental Status Examination; SGDS-K: Short-Form Geriatric Depression Scale-K; GQOL-D: Geriatric Quality of Life-Dementia.

## Data Availability

(1) The data used to support the findings of this study were supplied under license and so cannot be made freely available. Requests for access to these data should be made at dj7407@cju.ac.kr. (2) The data used to support the findings of this study are currently under embargo while the research findings are commercialized. Requests for data, 12 months after publication of this article, will be considered by the corresponding author. (3) The data used to support the findings of this study may be released upon application to Dr. Kim, who can be contacted at dj7407@cju.ac.kr.

## References

[1] Korea S. (2014). *Senior Statistics*.

[2] Park Y.-S., Jee Y.-J., Bae S.-H. (2017). Effects of a dementia family support program on families’ attitude towards dementia, desire for institutionalization, caregiving behavior and caregiving burden. *Asia-Pacific Journal of Multimedia Services Convergent with Art, Humanities, and Sociology*.

[3] Korea Central Dementia Center (2017). National Institute of Dementia. annual report. *Korean dementia observatory 2017*.

[4] Galimberit D., Scarpini E. (2011). Progress in Alzheimer's disease. *Journal of Neurology, Neurosurgery & Psychiatry*.

[5] Koo S. K., Park H. Y., Park J. H. (2017). A systematic review of nonpharmacological interventions on activities of daily living with dementia. *The Korean Gerontological Society*.

[6] Han Y. R., Song M. S., Lim J. Y. (2010). The effects of a cognitive enhancement group training program for community-dwelling elders. *Journal of Korean Academy of Nursing*.

[7] Kim K.-J., Noh D.-h., Han S.-H., Cha Y.-J., Kam K.-Y. (2020). Correlation between depression and memory according to apolipoprotein E genotype in elderly with Alzheimer's dementia. *The Korea Academia-Industrial cooperation Society*.

[8] Kim H. J., Song Y. H. (2014). The effect of reminiscence-oriented sand play for ego-integrity of the elderly. *Korean Journal of Play Therapy*.

[9] Lim S.-h., Shin J.-i. (2019). A systematic review of reminiscence therapy interventions applied to patients with in dementia. *Society of Occupational Therapy for the Aged and Dementia*.

[10] Wang J. J., Yen M., OuYang W. C. (2009). Group reminiscence intervention in Taiwanese elders with dementia. *Archives of Gerontology and Geriatrics*.

[11] Yim K. M., Park J. H. (2013). The effects of occupational reminiscence therapy applied to the elderly with dementia: a pilot study. *Journal of Korean Society of Occupational Therapy*.

[12] Park J. K., Kim J. M., Nam H. J. (2011). The effects of group reminiscence counseling on ego integrity, depression, death anxiety of the elderly. *Journal of the Korea Gerontological Society*.

[13] Lee J. H. (2016). The effect of shelter of dementia on cognitive function and depression level in elderly people in community. *The Journal of Occupational Therapy for the Aged and Dementia*.

[14] Kim S. Y., Kim Y. S., Nam Y. H., Kim H. (2018). Task-oriented approach program on self-efficacy scale and activities of daily living, geriatric quality of life in dementia patients. *Society of Occupational Therapy for the Aged and Dementia*.

[15] Jung B. H. (2013). The effect of cognitive occupational therapy in community living elders with mild cognitive impairment and dementia. *The Society of Digital Policy and Management*.

[16] Shin S. J., Lee J. S. (2013). The impact of cognitive behavioral therapy on wandering and care burdens for patient with dementia. *The Journal of Occupational Therapy for the Aged and Dementia*.

[17] Yim J. Z. (2010). Cognitive rehabilitation “dementia prevention program” operation case. *Korean Academy of Rural Health Nursing*.

[18] Ewing D. M. (1992). *Future competencies needed in the preparation of secretaries in the State of Illinois using the Delphi technique*.

[19] Hamilton B. B., Granger C. V., Sherwin F. S., Zielezny M., Tashman J. S. (1987). *A uniform national data system for medical rehabilitation*.

[20] Kang Y. W., Na D. R., Han S. H. (1997). A validity study on the Korean mini-mental state examination (K-MMSE) in dementia patients. *Journal of the Korean Neurological Association*.

[21] Han T. R., Shin H. I., Kim S. J. (2011). The effect of prism treatment in patients with stroke with unilateral ignorance - preliminary study. *Annals of Rehabilitation Medicine*.

[22] Lee J. S., Lee W. K., Park E. M. (2006). Subjective memory complaints and objective cognitive functions of the elderly living in the institution. *Korean Journal Psychopathology*.

[23] Yesavage J. A., Brink T. L., Rose T. L. (1982). Development and validation of a geriatric depression screening scale: a preliminary report. *Journal of Psychiatry Research*.

[24] Sheikh V. I., Yesavage V. A., Brink T. L. (1986). Geriatric depression scale (GDS): recent evidence and development of shorter version. *Clinical Gerontology; a Guide to Assessment and Intervention*.

[25] Cho M. J., Bae J. N., Suh G. H. (1999). Validation of Geriatric Depression Scale, Korean version (GDS) in the assessment of DSM-III-R major depression. *Journal of the Korean Neurological Association*.

[26] Kim M. J., Oh M. (2018). The effect of reminiscence-oriented group arts therapy on the ego integrity, depression and quality of life for the elderly patients with dementia in a nursing home. *Journal of Arts Psychotherapy*.

[27] Huang S.-L., Li C.-M., Yang C.-Y., Chen J.-J. J. (2009). Application of reminiscence treatment on older people with Dementia. *Journal of Nursing Research*.

[28] Eom M. Y. (2000). In a social welfare center for the elderly = evaluating the effects of a reminiscence group therapy for the elderly with stroke. *Journal of the Korea Gerontological Society*.

[29] Joo A. R., Park S. H. (2004). The relationship between cognitive function and depression in elderly people in rural areas. *Journal of Korean Gerontology Nurse*.

[30] Kim S. Y., Kim Y. S. (2014). Effects of occupational reminiscence therapy applied on depression, memory and behavioral problems in the elderly with dementia. *The Journal of Occupational Therapy for the Aged and Dementia*.

[31] Banerjee S., Smith S. C., Lamping D. L. (2006). Quality of life in dementia: more than just cognition. An analysis of associations with quality of life in dementia. *Journal of Neurology, Neurosurgery & Psychiatry*.

[32] Butler R. N. (2016). The life review: an interpretation of reminiscence in the aged. *Psychiatry*.

[33] Ha H. M., Choi E. Y., Jeon J. K. (2012). Effects of group art treatment reflecting reminiscence therapy on the quality of life of the elderly with mild dementia living in health care centers. *Korean Journal of Art Therapy*.

[34] Kim H. N., Park W. K. (2015). The effects of horiticultural activities program on depression of the dementia elderly at the facility. *Journal of Korean Aging Friendly Industry*.

[35] Chung J. C. C. (2016). Activity participation and well-being of people with dementia in long-term-care settings. *OTJR: Occupation, Participation and Health*.

